# Clinically interpretable electrovectorcardiographic machine learning criteria for the detection of echocardiographic left ventricular hypertrophy

**DOI:** 10.1371/journal.pone.0334829

**Published:** 2025-10-17

**Authors:** Fernando De la Garza-Salazar, Brian Egenriether

**Affiliations:** 1 Independent Researcher, Monterrey, México,; 2 Tecnológico de Monterrey, Escuela de Medicina, Avenida Ignacio Morones Prieto, Sertoma, Monterrey, Nuevo León, México,; 3 Independent Researcher, Charleston, South Carolina, United States of America; Albert Einstein College of Medicine, UNITED STATES OF AMERICA

## Abstract

Echocardiographic left ventricular hypertrophy (Echo-LVH) is frequently underdetected by traditional electrocardiogram (ECG) criteria due to limited sensitivity. We investigated whether integrating ECG with vectorcardiography (VCG) using a clinically interpretable machine learning algorithm (C5.0) could improve diagnostic performance. We analyzed ECG and VCG data from 664 patients, 42.8% of whom had Echo-LVH. The study introduced three new criteria—Marcos VCG, Marcos VCG-ECG, and Marcos VCG-ECGsp—named in honor of the software used for VCG synthesis, and compared their diagnostic performance against 23 established ECG criteria, including Cornell voltage, Peguero-Lo Presti, and Sokolow-Lyon. Marcos VCG-ECGsp, optimized for higher specificity, was included to evaluate trade-offs in performance. Validation was performed using train/test split and 10-fold cross-validation. Marcos VCG-ECG achieved higher AUC than Cornell voltage in both training (0.81 vs. 0.68, p < 0.0001) and testing (0.78 vs. 0.69, p = 0.04). The new criteria also showed superior sensitivity compared to Peguero-Lo Presti, the most sensitive traditional criterion (73.1%, 62.4%, 55.9% vs. 30.1%, p < 0.0001). While specificity was lower than Cornell (81.1% vs. 96.4%, p = 0.017), it remained acceptable, reflecting a clinically relevant trade-off favoring detection over false positives. In conclusion, integrating ECG with VCG through machine learning enances Echo-LVH detection, delivering superior sensitivity while preserving specificity. The proposed criteria are clinically interpretable, highlight the novelty of combining two electrical spectra, and hold potential to impact routine diagnostic practice.

## Introduction

Echocardiographic left ventricular hypertrophy (Echo-LVH) significantly predicts cardiovascular morbidity and mortality [1]. Electrocardiographic detection of LVH dates back to Einthoven’s initial descriptions in 1906 [2] and was further refined by criteria such as Cornell voltage (1985) [3] and Peguero-Lo Presti (2017) [4]. Despite decades of development [5], conventional ECG criteria still demonstrate poor sensitivity for Echo-LVH [6].

Vectorcardiography (VCG) [7], expanded electrocardiography by enabling three-dimensional representations of cardiac electrical activity [8,9]. Although early adoption was limited by hardware constraints [10], VCG synthesis via matrix multiplication became feasible with digital ECG systems in the 1980s [11–13]. Techniques such as the Inverse Dower matrix [11], Kors regression [12], and least-square VCG estimations (QLSV, PLSV) [13,14] have since facilitated advanced wave-specific analysis.

We recently developed *Marcos*, a VCG analysis software capable of synthesizing VCG using four matrix methods, quantifying global and intra-wave metrics (P, QRS, and T loops), and characterizing electrical loop morphology [15]. VCG metrics have shown diagnostic value in a variety of cardiovascular conditions, including ischemic heart disease, ventricular arrhythmias, cardiac resynchronization therapy, and myocardial infarction [16–24]. However, several VCG-derived metrics, particularly intra-waveform and loop morphology features, remain underexplored in the context of Echo-LVH detection.

Machine learning (ML) offers promising advances in electrodiagnostics [25,26]. Diverse algorithms such as SVM, Random Forest, GLMNet, XGBoost, AdaBoost, and various neural networks, have achieved sensitivities ranging from 29% to 96.6% [26]. However, most models incorporate non-electrical data and operate as black-box systems, which limits their interpretability and application in purely electrical diagnostics [26].

The C5.0 algorithm, a transparent, white-box ML model evolved from decision trees, allows interpretable rule-based classification [27]. Previously, we applied C5.0 to 31 manually extracted ECG features, producing a model with 71.4% accuracy, 79.6% sensitivity, and 53% specificity [28]. An automated version using 458 ECG parameters achieved 70.5% accuracy, 74.3% sensitivity, and 68.7% specificity using only three ECG predictors [29].

We hypothesize that integrating VCG-derived parameters into an interpretable C5.0 machine learning model would improve the diagnostic accuracy and sensitivity for detecting Echo-LVH compared to conventional ECG criteria, while maintaining acceptable levels of specificity. The model was designed to rely exclusively on electrical signals (ECG and VCG) without the inclusion of demographic or clinical variables.

This study presents the development and validation of the Marcos VCG, a model based solely on vectorcardiographic features; Marcos VCG-ECG, which integrates both vectorcardiographic and electrocardiographic variables; and Marcos VCG-ECGsp, optimized for higher specificity. Using the C5.0 algorithm and an extensive pool of quantitative ECG/VCG parameters, these models were developed with the goal of improving upon conventional ECG criteria in the detection of Echo-LVH, while prioritizing interpretability and consistent diagnostic performance

## Methods

### Study design and population

This retrospective, single-center study aimed to develop and internally validate a machine learning–based diagnostic prediction model for Echo-LVH. The analysis included adult patients who underwent both transthoracic echocardiography and 12‑lead ECG within seven days at the Cardiology Department of a tertiary hospital in Monterrey, Mexico, between 1 January 2016 and 31 August 2019. Ethical approval was obtained (CMHAE‑001‑19) with a waiver of individual consent, and the study adheres to the Declaration of Helsinki as well as STARD 2015 guidelines for diagnostic studies [30] and international guidelines for the development of machine learning models [31].

From 7 567 consecutive examinations, exclusions for age < 18 years, incomplete imaging, or predefined rhythm/structural abnormalities (full list in S1 Text) left 664 analysable patients. Clinical demographics and comorbidities (age, sex, BMI, BSA, hypertension, diabetes, etc.) were abstracted from electronic records; BSA was calculated with the Mosteller formula [32,33].

### Imaging and signal acquisition

Transthoracic echocardiographic studies were acquired on Philips EPIQ7/IE33 scanners using 2‑D–guided M‑mode in accordance with ASE/EACVI standards [33]. End‑diastolic left ventricular internal diameter (LVID), interventricular septal thickness (IVST) and left ventricular posterior wall thickness (LVPWT) were measured, and left‑ventricular mass (LVM), mass index (LVMI) and relative wall thickness (RWT) were calculated using guideline formulas. LVH was defined as LVMI > 115 g/m² in men or > 95 g/m² in women [26,33]. Three cardiologists performed the readings with excellent agreement (κ = 0.91). Segmental hypokinesia or akinesia on echo were noted as echo‑detected ischaemic heart disease (IHD).

Standard 12‑lead ECGs were recorded on a Philips PageWriter TC50 (10 mm/mV, 25 mm/s). The built‑in DXL‑16 algorithm automatically extracted 458 quantitative parameters per tracing; the full list is provided in S1 Table.

For comparison with established electrocardiographic markers of LVH, 23 contemporary ECG criteria—including Cornell voltage, Peguero‑Lo Presti and Sokolow‑Lyon—were computed. Voltage‑duration‑product variants of each criterion were also generated to allow sensitivity analyses.

Digital 12‑lead ECGs were transformed into orthogonal X‑, Y‑, Z loops using the Marcos software [15], which applies four validated transformation matrices. From each segmented P, QRS and T loop, 3 360 quantitative features were extracted, including isochronal velocity, angle and magnitude metrics [18–21,23,24,34–38]. Full details on the transformation, segmentation and feature extraction pipeline have been reported previously [15] and are available in Supplementary S2 Text

### Feature extraction and data preprocessing

Angular variables were expressed in radians; missing values were imputed (mean for symmetric, median for skewed distributions), and all continuous predictors were z‑score scaled to minimise unit‑driven bias. Models were trained on the standardised data, but decision‑tree cut‑offs are reported in original units for clinical interpretability.

Records were randomly allocated 70% to a training set (n = 460) and 30% to a test set (n = 204); age, sex, comorbidities and Echo‑LVH distribution were comparable between sets (all p > 0.05; Table 1).

**Table 1:** Demographic and echocardiographic characteristics of the study population.

	Total (n = 664)	Training set (n = 460)	Testing set (n = 204)	p-value
**Demographics**				
Age (years) [mean, SD]	64.2 (15.1)	64.4 (15)	63.8 (15.3)	0.67
Body surface area (m^2^) [mean, SD]	1.9 (0.24)	1.9 (0.25)	1.9 (0.25)	0.96
Body mass index (kg/m^2^) [mean, SD]	28 (5.2)	28.1 (5.1)	27.8 (5.2)	0.36
Sex (female/male) (n, %)	285 (42.9)/ 379 (57.1)	194 (42.2)/ 266 (57.8)	91 (44.6)/ 113 (55.4)	0.56
**Echocardiographic findings**				
Echo-LVH (n, %)	284 (42.8)	191 (41.5)	93 (45.6)	0.33
IHD on echo (n, %)	116 (17.5)	74 (16.1)	42 (20.6)	0.19
Echo-LVH severity (n, %)				0.80
Mild	108 (16.3)	73 (15.9)	35 (17.2)	
Moderate	66 (9.9)	44 (9.6)	22 (10.8)	
Severe	110 (16.6)	74 (16.1)	36 (17.6)	
LV geometry (n, %)				0.75
Normal	146 (22)	105 (22.8)	41 (20.1)	
Concentric remodeling	233 (35.1)	163 (35.4)	70 (34.3)	
Concentric hypertrophy	232 (34.9)	155 (33.7)	77 (37.7)	
Eccentric hypertrophy	53 (8)	37 (8)	16 (7.8)	
**Comorbidities**				
Atrial fibrillation on ECG (n, %)	52 (7.8)	41 (8.9)	11 (5.4)	0.12
Chronic heart failure (n/N, %)^‡^	50/482 (10.4)	35/337 (10.4)	15/145 (10.3)	0.989
Chronic kidney disease (n/N, %)^‡^	39/482 (8.1)	27/337 (8)	12/145 (8.3)	0.92
Diabetes (n/N, %)^‡^	147/482 (30.5)	107/337 (31.8)	40/145 (27.6)	0.36
Dyslipidemia (n/N, %)^‡^	75/482 (15.6)	47/337 (13.9)	28/145 (19.3)	0.14
History of IHD (n/N, %)*	213/491 (43.4)	149/342 (43.6)	64/149 (43)	0.90
Hypertension (n/N, %)^†^	281/486 (57.8)	192/340 (56.5)	89/146 (61)	0.36
Stroke (n/N, %)^‡^	46/482 (9.5)	35/337 (10.4)	11/145 (7.6)	0.38

Baseline demographic, echocardiographic and comorbidity profile of the training and test cohorts. Values are mean ± SD for continuous variables and n (%) for categorical variables. P values correspond to t‑test (Age, BMI) applied on log‑transformed data when appropriate or χ² (categorical variables). Valid n (train/test): *342 and 149,

† 340 and 146,

‡ 337 and 145,

§ 265 and 104 for the comorbidities indicated. Abbreviations: Echo‑LVH, echocardiographic left‑ventricular hypertrophy; IHD, ischemic heart disease; LV, left ventricle.

High‑dimensional feature spaces were pruned with Lasso regression applied separately to VCG and ECG variables [39,40], retaining the subset that maximised cross‑validated AUC in the training data.

### Model development and evaluation

Feature‑reduced datasets were modelled with the white‑box C5.0 decision‑tree algorithm [41]. Separate C5.0 models were trained using: (i) VCG predictors alone, (ii) combined VCG + ECG predictors, and (iii) a variant tuned for higher specificity via cost‑matrix weighting. Hyperparameters (minCases, winnowing, cost matrix) were optimised by grid search within the training set; trees were pruned automatically to prevent over‑fitting [42]. A fixed probability threshold of 0.50 defined Echo‑LVH presence, consistent with previous work [28,29]. Final models were selected based on training‑set AUC and rule simplicity and subsequently assessed on the independent test set.

### Statistical analysis

Continuous and categorical variables were summarized as mean ± SD and n (%), respectively. Normality was assessed using the Kolmogorov–Smirnov test. Group differences between training and test sets were evaluated using Student’s t-test for continuous variables and χ² or Fisher’s exact test for categorical variables, as appropriate; continuous variables were log-transformed when normality assumptions were not met.

Model performance for Echo‑LVH detection was evaluated using AUC (pROC package) with DeLong comparisons [43,44]. Accuracy, sensitivity, specificity, positive predictive value (PPV), negative predictive value (NPV), and F1‑score were computed from confusion matrices [45,46]. Traditional ECG criteria were compared using McNemar’s test with Bonferroni correction [45].

Internal validation used 10‑fold cross‑validation within the training set [47]. Subgroup analyses considered sex, age > 60 years, comorbidities (e.g., hypertension, obesity), Echo‑LVH geometry, and severity grades.

Correct classification rates (CCR) were reported for each left ventricle geometry and LVH severity category. All analyses were conducted in RStudio (C50, ggplot2) [27,48], with p < 0.05 considered significant.

### Sample size determination and data integrity

An *a priori* power analysis (R pwr package) indicated that ≥ 178 Echo‑LVH cases, and 178 controls would provide 80% power (α = 0.05) to detect a 10‑percentage‑point improvement in sensitivity over conventional ECG criteria (30% to 40%) [49].

## Results

### Baseline characteristics

A total of 664 patients were analyzed, with 42.8% (n = 284) presenting Echo-LVH. Table 1 compares demographic and echocardiographic data between the training (n = 460) and testing (n = 204) sets. Both cohorts had similar Echo-LVH prevalence, LV geometries, and comorbidity distribution, supporting sample comparability (Table 1).

### Model development and performance

Lasso regression identified 33 VCG and 26 ECG variables as relevant (S4 Table). The final Marcos decision tree models (C5.0) selected 11 of these as core predictors (Fig 2 and Table 2).

**Table 2:** Description of electrocardiographic and vectorcardiographic features relevant to Echo-LVH detection.

Predictor No.	Abbreviation [Unit]	Description
**Marcos VCG (VCG data only, 5 predictors)**		
1	GAV_K [None]	Geometric area vector of QRS loop (Kors)
2	RangeAngR [°]	Rotational angle range of QRS loop (Dower)
3	PMagP2 [μV]	Magnitude in second decile of P loop (PLSV)
4	POrbFrqD5 [ms ⁻ ¹]	Orbital frequency in fifth decile of P loop (Dower)
5	Vel Min Kf T [μV/ms]	Minimum velocity of T loop (Kors + Fourier)
**Marcos VCG-ECG (VCG and ECG data, 6 predictors)**		
1	Ang Term Num [mV]	Magnitude of terminal QRS vector
2	T AREA DI [Ashman Units]*	Area under T wave in lead DI
3	ECG 21 [mV]	Cornell voltage (RaVL + SV3)
4	QRSMagP1 [μV]	Magnitude in first decile of QRS loop (PLSV)
5	TVelQ5 [μV/ms]	Velocity in fifth T loop decile (QLSV)
6	QRSMagPf9 [μV]	Magnitude in ninth decile of QRS loop (PLSV + Fourier)

Key VCG and ECG predictors used in the Marcos decision trees, with units and brief functional descriptions. *One Ashman Unit = 40 msec x 0.1 mV

**Fig 1:**
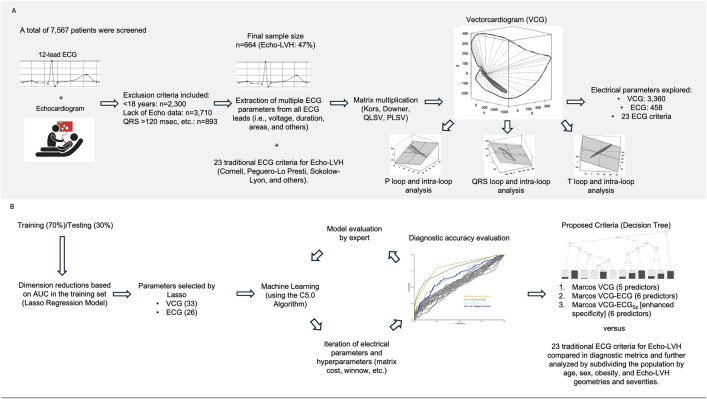
Methodological Flowchart for Enhanced Echo-LVH Detection. Fig 1 shows the methodological approach for detecting Echo-LVH. **(A)** Upper panel describes the patient cohort selection and extraction of VCG and ECG parameters. **(B)** Bottom panel outlines the use of statistical and machine learning techniques, including Lasso regression and the C5.0 algorithm, to develop the Marcos VCG and Marcos VCG-ECG criteria. Abbreviations: C5.0: Decision Tree Machine Learning Algorithm, ECG: Electrocardiography, Echo-LVH: Echocardiographic Left Ventricular Hypertrophy, LASSO: Least Absolute Shrinkage and Selection Operator, VCG: Vectorcardiography.

The Marcos VCG model, based on five vectorcardiographic features, includes atrial and ventricular depolarization and repolarization metrics. It achieved an internal validation accuracy of 76.1% (95% CI: 61.3%–87.3%), with sensitivity of 59.6%, specificity of 87.8%, PPV of 78.2%, and NPV of 75.5%. These results were consistent in both the training (accuracy 76.1%, F1-score 0.674) and testing sets (72.1%, F1-score 0.645) (Table 3).

**Table 3:** Performance metrics of the Marcos models in training and testing sets.

Model	Acc (95% CI)	Sensitivity (%)	F1-score	Specificity (%)	PPV (%)	NPV (%)
**Training set (n = 460)**						
Marcos VCG	76.1 (71.9-79.9)	59.7	0.674	87.7	77.6	75.4
Marcos VCG-ECG	76.7 (72.6-80.5)	77.5	0.734	76.2	69.8	82.7
Marcos VCG-ECGsp	75.4 (71.2-79.3)	63.4	0.682	84	73.8	76.4
**Testing set (n = 204)**						
Marcos VCG	72.1 (65.4-78.1)	55.9	0.645	85.6	76.5	69.9
Marcos VCG-ECG	75.5 (69-81.2)	73.1	0.731	77.5	73.1	77.5
Marcos VCG-ECGsp	72.6 (65.9-78.6)	62.4	0.674	81.1	73.4	72

Performance Metrics of Marcos VCG, Marcos VCG-ECG and Marcos VCG-ECGsp Criteria. This table presents the performance metrics of Marcos criteria in both training and testing sets, including key statistical metrics. The highest-performing model in each category is indicated by gray cell shading in the table. Abbreviations: Acc = accuracy; PPV = positive predictive value; NPV = negative predictive value.

Similarly, the Marcos VCG-ECG model, combining six ECG and VCG variables, showed improved diagnostic balance by jointly capturing depolarization and repolarization patterns. It yielded an accuracy of 76.7% (95% CI: 61.2–87.9%) in internal validation, with high sensitivity (77.4%) and NPV (83.3%). Its performance remained robust in training (F1-score 0.734) and testing (F1-score 0.731) cohorts (Table 3).

The simplified Marcos VCG-ECGsp model demonstrated an internal accuracy of 75.4% (95% CI: 60.6–86.8%), sensitivity of 63.3%, and specificity of 84% (S5 Table). While slightly reducing sensitivity (63.3% and 63.4%) in favor of specificity (84% and 81.1%), its performance remained consistent across training (75.4%) and testing (72.6%) sets (Table 3). This model may be particularly useful when the clinical priority is reducing false positives.

### Head‑to‑head comparison with ECG criteria

The Marcos criteria outperformed 23 classical ECG models in diagnostic accuracy, sensitivity, and F1-score (Fig 3, Table 4). The Marcos VCG-ECG criteria achieved higher AUC than the Cornell voltage criterion in both training (0.81 vs. 0.68, p < 0.0001) and testing (0.78 vs. 0.69, p = 0.04). Compared to our approach, classic ECG models showed 11.8%–27.2% lower accuracy, 43%–73.1% lower sensitivity, and F1-score reductions of 0.298–0.71 (S7 Table).

**Table 4:** Diagnostic performance of Marcos models versus classic ECG criteria in test set.

Criteria [# predictors]	Acc (95%CI)	Δ Acc (%)	Se (%)	Δ Se (%)	F1 score	Sp (%)	PPV (%)	NPV (%)
Marcos VCG-ECG [6]	75.5 (69-81.2)	Baseline	73.1	Baseline	0.731	77.5	73.1	77.5
Marcos VCG-ECGsp [6]	72.6 (65.9-78.6)	−2.9	62.4	−10.7	0.674	81.1	73.4	72
Marcos VCG [5]	72.1 (65.4-78.1)	−3.4	55.9	−17.2	0.645	85.6	76.5	69.9
Cornell voltage [2]^†^	62.3 (55.2-68.9)	−13.2	21.5	−51.6	0.341	96.4	83.3	59.4
Peguero-Lo Presti [13]^‡^	63.7 (56.7-70.3)	−11.8	30.1	−43	0.433	91.9	75.7	61.1

Diagnostic performance in the test set: Marcos models versus the two leading ECG criteria (Cornell voltage, Peguero‑Lo Presti). Δ values indicate the difference from the reference model (Marcos VCG‑ECG). All Marcos models outperform the ECG benchmarks across key indices. Δ values are differences versus Marcos VCG‑ECG.

† Cut-off: > 2.8 mV (men)/ > 2.0 mV (women).

‡ Cut-off: ≥ 2.8 mV (men)/ ≥ 2.3 mV (women). The highest-performing model in each category is indicated by gray cell shading in the table. Abbreviations: Acc: accuracy, Se: Sensitivity, Sp: Specificity, PPV: Positive Predictive Value, NPV: Negative Predictive Value.

**Fig 2:**
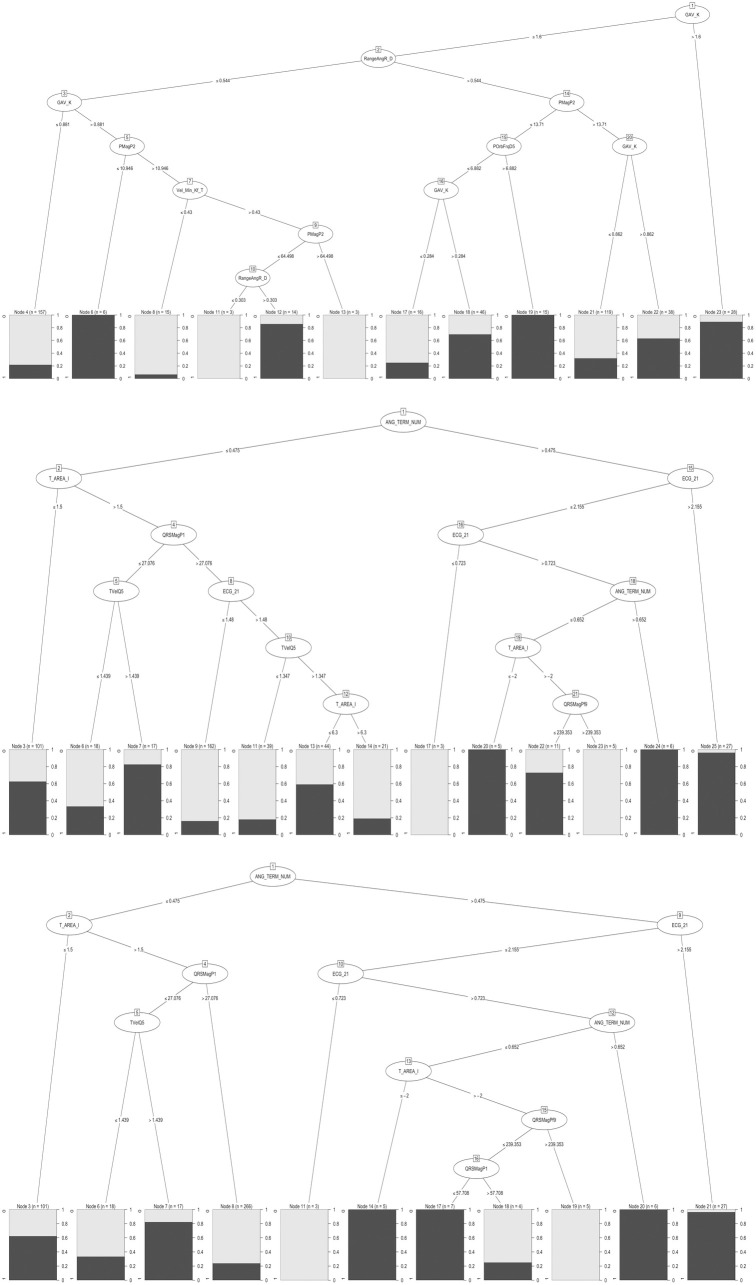
Decision tree algorithms for Echo-LVH detection using Marcos VCG and ECG criteria. C5.0 decision trees for **(A)** Marcos VCG, **(B)** Marcos VCG‑ECG and **(C)** Marcos VCG‑ECGsp. Bar height at each leaf indicates the number of patients classified as Echo‑LVH (dark) versus non‑LVH (light). Abbreviations: ANG_TERM_NUM: Magnitude of the ventricular depolarization terminal vector (mV), ECG 21: Cornell voltage criteria (mV), GAV_K: Geometric area vector of ventricular depolarization (None), PMagP2: Magnitude of auricular depolarization near the onset (2nd part of the loop) (μV), POrbFrqD5: Orbital frequency of auricular depolarization in the middle (5th part of the loop) (ms − 1), QRSMagP1: Ventricular depolarization magnitude at the onset (first part of the loop) (μV), QRSMagPf9: Ventricular depolarization magnitude at the offset (9th part of the loop) (μV), RangeAngR: Rotational angle range of ventricular depolarization (°), T AREA DI: T wave area in DI (Ashman Units), TVelQ5: Ventricular repolarization velocity in the middle of the loop (μV/ms), Vel Min Kf T: Minimum velocity of ventricular repolarization (μV/ms).

**Fig 3:**
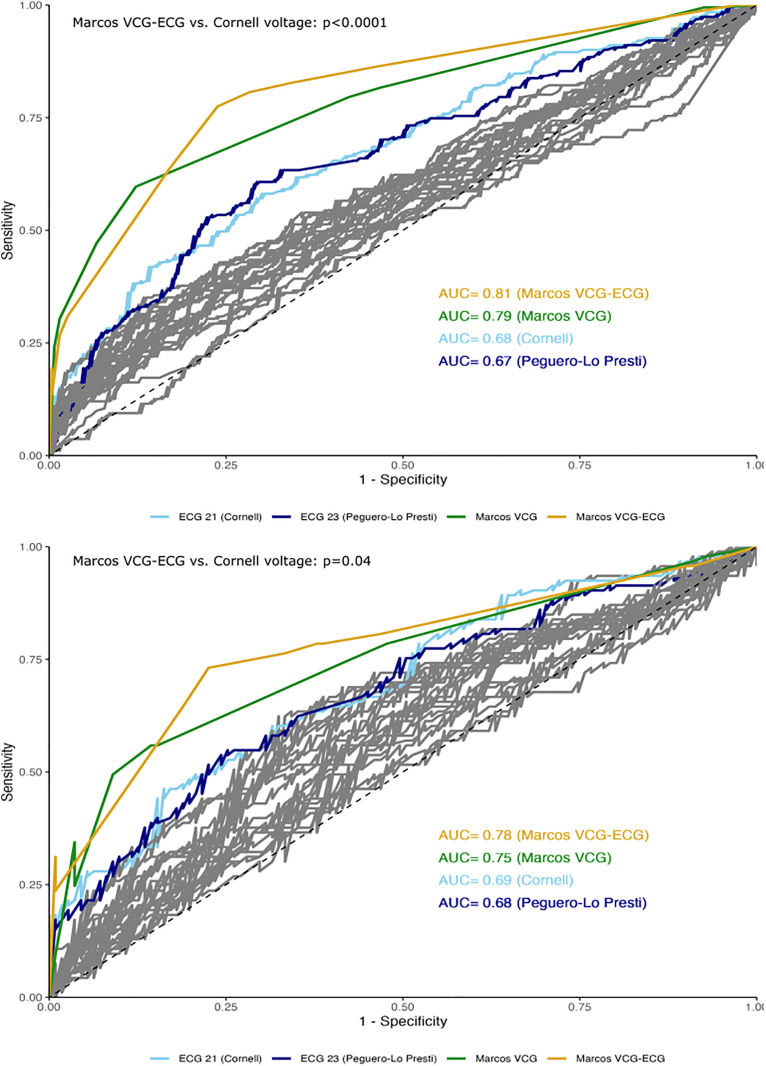
Receiver Operating Characteristic (ROC) curves comparison. ROC curves for Marcos VCG‑ECG versus the best conventional criterion (Cornell voltage) in the training cohort (A, n = 460) and the independent test cohort (B, n = 204). Marcos VCG‑ECG achieved a significantly higher AUC in both sets (DeLong p < 0.0001 and p = 0.04). Abbreviations: AUC: Area Under the Curve, ROC: Receiver Operating Characteristic, ECG: Electrocardiography, Echo-LVH: Echocardiographic Left Ventricular Hypertrophy, VCG: Vectorcardiography.

**Fig 4:**
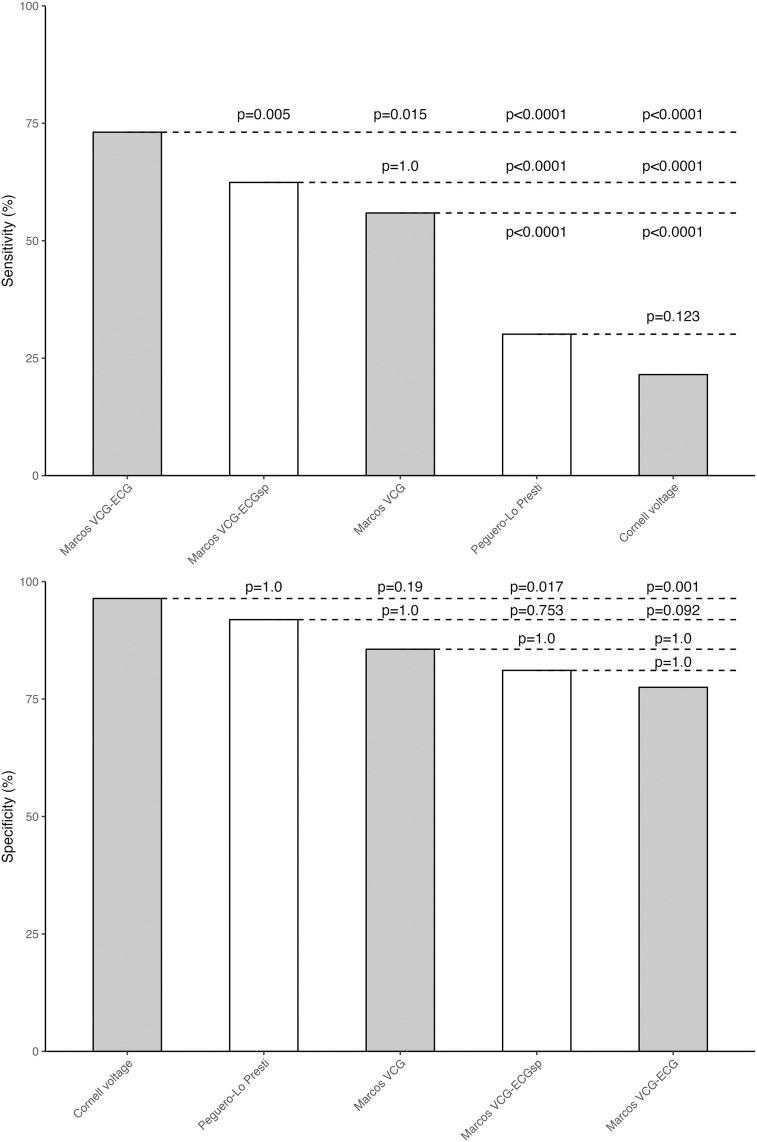
Sensitivity and specificity comparisons across the proposed criteria and the most sensitive ECG criteria. Comparative bar plots of sensitivity (A) and specificity (B) for Marcos models versus the two most sensitive ECG criteria. P values were obtained with the McNemar test and adjusted using Bonferroni correction for multiple comparisons; adjusted values were truncated to 1.0 when exceeding that threshold.

The VCG-ECG model had the highest sensitivity and F1-score overall, significantly outperforming the VCG-only, VCG-ECGsp, Cornell voltage, and Peguero-Lo Presti criteria (all p < 0.05 for sensitivity) (Fig 4). Cornell voltage was more specific than VCG-ECG and VCG-ECGsp, but not VCG (p = 0.19). Peguero showed no significant specificity advantage over any Marcos model.

In summary, the Marcos criteria substantially improve sensitivity while maintaining specificity within acceptable ranges.

### Subgroup analyses

The proposed models maintained consistent performance across key subgroups (Table 5). The VCG-only criteria demonstrated superior accuracy in females and patients with IHD on echo— populations where classic ECG criteria are known to have limitations. Although Cornell voltage and Peguero-Lo Presti showed high specificity (>90%), their sensitivity remained low across all subpopulations (15.6%–42.9%).

**Table 5:** Subanalysis of diagnostic performance of Marcos criteria in different populations in test set.

Subgroup	Metric	Marcos VCG	Marcos VCG-ECG	Cornell voltage	Peguero-Lo Presti
**Female (n = 91)**	Acc (95% CI)	74.7 (64.5–83.3)	74.7 (64.5–83.3)	57.1 (46.3–67.5)	60.4 (49.6–70.5)
	Se (%)	60.4	70.8	27.1	31.3
	Sp (%)	90.7	79.1	90.7	93
**Male (n = 113)**	Acc (95% CI)	69.9 (60.6–78.2)	76.1 (67.2–83.6)	66.4 (56.9–75.0)	67.3 (57.8–75.8)
	Se (%)	51.1	75.6	15.6	28.9
	Sp (%)	82.3	76.5	100	92.7
**>60 years (n = 127)**	Acc (95% CI)	69.3 (60.5–77.2)	71.7 (63.0–78.3)	57.6 (48.4–66.2)	60.6 (51.6–69.2)
	Se (%)	52.4	74.6	20.6	27
	Sp (%)	85.9	68.8	93.8	93.8
**≤60 years (n = 77)**	Acc (95% CI)	76.6 (65.6–85.5)	81.8 (71.4–89.7)	70.1 (58.6–80.0)	70.1 (58.6–80.0)
	Se (%)	63.3	70	23.3	36.7
	Sp (%)	85.1	89.4	100	91.5
**BMI ≥ 30 kg/m² (n = 54)**	Acc (95% CI)	64.8 (50.6–77.3)	72.2 (58.4–83.5)	55.6 (41.4–69.1)	57.4 (43.2–70.8)
	Se (%)	44.8	72.4	17.2	27.6
	Sp (%)	88	72	100	92
**BMI < 30 kg/m² (n = 150)**	Acc (95% CI)	74.7 (66.9–81.1)	76.7 (69.1–83.2)	64.7 (56.5–72.3)	66.7 (58.5–74.1)
	Se (%)	60.9	73.4	23.4	31.3
	Sp (%)	84.9	79.1	95.4	93
**Hypertension (n = 89)**	Acc (95% CI)	66.3 (55.5–76.0)	71.2 (61.4–80.9)	51.7 (40.8–62.4)	58.4 (47.5–68.8)
	Se (%)	50.9	75.5	22.6	35.9
	Sp (%)	88.9	66.7	94.4	91.7
**IHD on echo (n = 42)**	Acc (95% CI)	78.6 (63.2–89.7)	69.1 (52.9–82.4)	61.9 (45.6–76.4)	71.4 (55.4–84.3)
	Se (%)	66.7	81	23.8	42.9
	Sp (%)	90.5	57.1	100	100

This table presents a detailed subanalysis of the Marcos criteria’s performance across various patient subgroups. The highest-performing model in each category is indicated by gray cell shading in the table. Abbreviations: Acc: accuracy, BMI: body mass index, IHD: ischemic heart disease, Se: Sensitivity, Sp: Specificity.

In geometric classification (Table 6), Cornell voltage and Peguero-Lo Presti achieved higher CCR for normal geometry (+24.4% and +14.6%) and concentric remodeling (+15.7% each), reflecting their conservative profile. Despite this, Marcos VCG maintained high CCRs: 82.9% (normal geometry), 87.1% (concentric remodeling), and 85.6% (normal LVMI).

**Table 6:** Correct classification rate of best criteria in test set.

	Marcos VCG-ECG (CCR, %) [baseline]	Marcos VCG (CCR, %)	Δ CCR (%)*	Cornell voltage (CCR, %)	Δ CCR (%)**	Peguero-Lo Presti (CCR, %)	Δ CCR (%)***
**Left ventricle geometry**							
Normal geometry (n = 41)	30 (73.2%)	34 (82.9%)	+9.7	40 (97.6%)	+24.4	36 (87.8%)	+14.6
Concentric remodeling (n = 70)	56 (80%)	61 (87.1%)	+7.1	67 (95.7%)	+15.7	67 (95.7%)	+15.7
Concentric hypertrophy ( = 77)	56 (72.7%)	43 (55.8%)	−16.9	17 (22.1%)	−50.6	23 (29.9%)	−42.8
Eccentric hypertrophy (n = 16)	12 (75%)	9 (56.3%)	−18.7	3 (18.8%)	−56.2	3 (18.8%)	−56.2
**Echo-LVH severity**							
Without Echo-LVH (n = 111)	86 (77.5%)	95 (85.6%)	+8.1	107 (96.4%)	+18.9	103 (92.8%)	+15.3
Mild Echo-LVH (n = 35)	27 (77.1%)	18 (51.4%)	−25.7	5 (14.3%)	−62.8	8 (22.9%)	−54.2
Moderate Echo-LVH (n = 22)	14 (63.6%)	15 (68.2%)	+4.6	6 (27.3%)	−36.3	7 (31.8%)	−31.8
Severe Echo-LVH (n = 36)	23 (63.9%)	19 (52.8%)	−11.1	9 (25%)	−38.9	11 (30.6%)	−33.3

Correct‑classification rate (CCR) of each criterion by LV geometry and Echo‑LVH severity in the test set. Gray shading marks the best model per row. Abbreviations: Echo‑LVH, echocardiographic left‑ventricular hypertrophy.

*Δ CCR vs Marcos VCG‑ECG: *Marcos VCG; **Cornell voltage; **Peguero‑Lo Presti.

The VCG-ECG model achieved the highest CCRs for pathologic patterns—concentric LVH (72.7%), eccentric LVH (75%), and all grades of severity: 77.1% (mild), 63.6% (moderate), and 63.9% (severe). In contrast, Cornell voltage and Peguero-Lo Presti underperformed with lower CCRs in these categories (Table 6).

## Discussion

### Main findings and diagnostic innovation

In this study, we developed three clinically interpretable C5.0 machine learning models that integrates VCG and ECG data without relying on demographic or clinical variables to enhance the detection of Echo-LVH (Fig 1). Remarkably, in specific groups such as patients without hypertension or those under 60 years old, the proposed criteria achieved accuracies exceeding 80% (Table 5 and S8 Table). Additionally, by performing a detailed analysis of P, QRS, and T loops—including intraloop dynamics— our approach offers a complementary diagnostic perspective that extends beyond current ECG or VCG criteria. These findings support our hypothesis that VCG and ECG integration into a interpretable model enhances sensitivity and diagnostic accuracy, while maintaining clinically acceptable specificity. Importantly, all predictions were generated through fully automated ECG and VCG interpretation, illustrating the potential of automated systems to support clinical decision-making [50,51].

### Advancing VCG interpretation

Early studies highlighted VCG’s ability to analyze complex QRS morphology, ST segments, T wave configurations, and QRS-T angles, demonstrating its diagnostic utility for Echo-LVH [52]. Subsequent research revealed significant changes in QRS loop forces and durations in patients with LVH, furthering our understanding of its pathophysiology [53]. Unlike previous studies that primarily focused on descriptive loop analysis, such as loop-specific configurations like loop folding, our research employs an automated, quantitative approach to VCG analysis [54]. Notably, a study showed that normal VCG variables were highly specific for normal LVM [55]. This aligns with our findings, where the VCG model demonstrated the highest specificity among the proposed criteria (Table 3) [55].

### A description of the Marcos criteria

The ventricular depolarization activation gradient, or GAV, consistently used in the VCG model (100% usage), indicates distortions like concavities previously identified in Echo-LVH cases [23]. Our findings show an increase in the minimum velocity of ventricular repolarization (Vel Min Kf T) in patients with Echo-LVH, aligning with research indicating that hypertrophied myocardium can exhibit a similar behavior during depolarization [56].

The Marcos VCG-ECG version exhibits improved accuracy (75.5%) over the VCG model (72.1%), with well-balanced sensitivity (73.1%) and specificity (77.5%) (McNemar test p-value: 0.51) (Figs 2 and 4, Table 3). The VCG-ECGsp criteria, streamlined for simplicity, offer enhanced specificity over the Marcos VCG-ECG model, albeit with a modest significant reduction in sensitivity (Fig 2 and Table 3). Integrating VCG and ECG metrics, particularly the Cornell voltage criterion, renowned for its efficacy in predicting major adverse cardiovascular events [57], illustrates a combined diagnostic approach that capitalizes on the complementary features of both signal types. Additionally, the inclusion of variables that explore the ventricular repolarization area (low area under T wave in lead DI), an early marker of repolarization alteration in patients with hypertension, underscores the potential in detecting hypertensive heart disease-related changes [58].

### Comparative value of VCG versus ECG criteria

Our analysis reveals that, compared to Peguero-Lo Presti and Cornell voltage, the VCG-ECG model exhibits higher accuracy (75.5%) and sensitivity (73.1%), although with slightly reduced specificity (Table 4 and Fig 4). Notably, the VCG-only version demonstrated significantly higher sensitivity than both Cornell and Peguero–Lo Presti criteria, while maintaining comparable specificity, suggesting it may serve as a useful complement to traditional ECG criteria in certain clinical contexts (Fig 4).

The first study of Cornell voltage criteria resulted in a sensitivity for Echo-LVH of 41%, specificity of 90%, and accuracy of 68% [3], while Peguero-Lo Presti’s first publication reported a sensitivity of 62% with a specificity of 90% [4]. However, subsequent data have shown highly variable results. For instance, a meta-analysis of over 13,000 patients revealed that the Peguero-Lo Presti and Cornell voltage criteria achieved accuracies and sensitivities of approximately 69% and 52% and 67% and 92%, respectively, aligning with our observations [59]. Other investigations have shown similar performance trends in older adult populations with Peguero-Lo Presti’s and the Sokolow-Lyon voltage index [60]. Another study noted Peguero-Lo Presti’s enhanced sensitivity at 55%, despite a specificity decrease to 72% [61]. A separate metanalysis reported Peguero-Lo Presti had the highest pooled sensitivity (43%) followed by Cornell voltage criteria (26.1%) and Sokolow-Lyon (22%) [62].

Research across diverse regions, including China and Brazil, supports these patterns, reporting comparable AUCs (0.6 and 0.69), accuracies (33.8% and 56.3%), sensitivities (15–31.9% and 6–41%), and specificities (>90% and >78.5%) across various ECG-based criteria [63,64]. Our sensitivity results align with prior reports ranging from 17.5% to 29.9% across various cohorts [65–68].

Recently, it has been acknowledged that the superiority of Peguero-Lo Presti for diagnosing Echo-LVH or predicting major cardiovascular outcomes over other ECG strategies “does not appear to be sufficiently proven” [69]. It is worth noting that a multivariable approach may offer advantages for Echo-LVH detection due to its capacity to integrate diverse electrical features, acknowledging the heterogeneity of electrocardiographic alterations in this condition (Fig 2) [70].

### Detection performance by Echo-LVH geometry

Moreover, our study demonstrates more consistent detection of both concentric and eccentric variants of Echo-LVH than standard ECG-based methods (Table 6). Previous studies have shown that Cornell voltage sensitivity for these geometries is 29.3% for concentric and 14.9% for eccentric with specificity at 96.5% for both [63]. Another analysis found that the accuracy of ECG in identifying concentric Echo-LVH ranged from 44.5% to 58.4%, with sensitivities as low as 5% to 33.9% and showed similarly limited performance in eccentric cases [64].

Additionally, another study in patients with essential hypertension using linear regression showed that RaVL + SD voltage in male subjects followed an increasing trend across the spectrum from normal geometry to concentric remodeling, concentric hypertrophy, and finally eccentric hypertrophy. However, traditional ECG metrics exhibited reduced performance compared to the VCG-ECG approach, which showed higher sensitivity for detecting both concentric (+50.6% and +42.8%) and eccentric (+56.2%) patterns (Table 6) [71].

### Performance across diverse subgroups

Our results demonstrate improved performance in subgroups where ECG-based detection is often limited, particularly in normotensive, younger, or obese individuals. Age, sex, obesity, hypertension, and myocardial ischemia can affect the performance of ECG criteria for Echo-LVH. A study on patients with hypertension reported lower sensitivities (10%−17.5%) compared to our findings [72]. In line with this, we observed that Cornell voltage and Peguero-Lo Presti showed reduced accuracy in hypertensive individuals (51.7% and 58.4%) versus non-hypertensive counterparts [72]. In contrast, the Marcos VCG-ECG criteria showed a higher accuracy in both groups: 71.2% and 82.5%, respectively (Table 5 and S8 Table).

Compared with normal-weight individuals, obese and overweight patients had lower Sokolow-Lyon voltage and a reduced prevalence of ECG-detected LVH using this criterion (31.4% versus 16.2% versus 10.9%, p < 0.001) [73]. Another report found that Cornell voltage sensitivity significantly declined in patients with BMI > 30 kg/m² relative to those with a BMI ≤ 25 kg/m² [74]. As a result, standard ECG benchmarks, often fail to achieve sufficient sensitivity (0–20%) in obese populations, even after BMI correction [75].

In patients with IHD on echo, loss of ventricular mass may result in voltage reduction, further diminishing ECG sensitivity [76]. In our study, IHD on echo adversely impacted the performance of all evaluated criteria except the VCG model, which maintained a high accuracy (78.6%) (Table 5).

### Comparison with other machine learning models

Several machine learning models have been proposed for the electrocardiographic detection of Echo-LVH, with varying degrees of performance depending on population characteristics, input variables, and model complexity (S9 Table) [77–87]. These approaches typically combine clinical, laboratory, and ECG parameters, and frequently utilize black-box algorithms such as random forests, convolutional neural networks, or ensemble methods. While some achieved high specificity or F1-scores, few provided a balanced combination of accuracy, sensitivity, and interpretability (S8 Table). Notably, only one study incorporated VCG and ECG for model construction, achieving a high F1-score but lacking detailed reporting of predictors [87]. In contrast, the Marcos criteria rely solely on ECG and VCG signals and require fewer inputs (S8 Table).

### Study limitations and methodological considerations

The retrospective and single-center nature of our study, alongside a modest cohort size, underscores inherent constraints, suggesting the preliminary nature of our results. Although internal validation was robust, no external validation was performed, and the applicability of the criteria to other populations remains to be established. In addition, the Marcos software used for VCG synthesis, while previously validated, is not yet widely available, which may limit immediate reproducibility.

The primary intention of this study is not to declare the proposed models as universally superior to ECG benchmarks but to introduce and evaluate a novel methodological framework: combining VCG synthesis, electrovectorcardiographic quantification, and interpretable machine learning for optimizing Echo-LVH detection. This work serves as a proof-of-concept exploration in a cohort of Mexican patients diagnosed with Echo-LVH.

Finally, patients with conditions potentially affecting ECG/VCG interpretation (e.g., bundle branch block, paced rhythms) were excluded, limiting generalizability in such scenarios.

Overall, the results are consistent with our hypothesis: integrating VCG with ECG using a white-box machine learning model like C5.0 improved diagnostic accuracy and showed a notable gain in sensitivity compared to traditional ECG criteria. Although specificity did not exceed that of the most specific ECG criterion (Cornell voltage), it remained within clinically acceptable ranges across all proposed models. These findings support the exploratory use implementation of electrovectorcardiographic decision trees in diagnostic workflows for Echo-LVH.

### Future directions and clinical translation

Building on these findings, future research will leverage publicly available datasets to externally validate optimized Marcos-based models across diverse populations and cardiovascular conditions. In addition, the predictive potential of the Marcos software might be explored beyond Echo-LVH by developing models to forecast major adverse cardiovascular events (MACE) and other cardiac conditions, including arrhythmic and structural heart diseases. Finally, further development of the software to incorporate additional electrophysiological spectra may help expand its diagnostic scope and potential translational utility.

## Conclusion

In conclusion, the detection of Echo-LVH through the integration of electrovectorcardiography and the ML C5.0 algorithm achieved higher sensitivity and overall accuracy than traditional ECG criteria, while maintaining clinically acceptable specificity. Among all models, the VCG-ECG model demonstrated the best overall performance. Additionally, the VCG model showed significantly higher sensitivity than both the Cornell voltage and Peguero–Lo Presti criteria, while preserving comparable specificity—highlighting its potential as a streamlined alternative in select clinical scenarios. Given its interpretability and diagnostic accuracy, the Marcos VCG-ECG criteria may be further explored for integration into ECG/VCG software to support LVH screening in clinical practice.

## Supporting information

S1 Table List of electrocardiogram (ECG) parameters analyzed by the Philips DXL-16 algorithm. (DOCX)

S2 Table Diagnostic criteria and corresponding ECG cut-values by various authors. (DOCX)

S3 Table Parameters of vectorcardiography (VCG) and their clinical descriptions. (DOCX)

S4 Table Low-prevalence comorbidities and conduction disorders in the training and test cohorts. (DOCX)

S5 Table Dimensionality reduction of VCG and ECG parameters with Lasso regression. (DOCX)

S6 Table Ten-fold cross validation for the proposed Marcos VCG, VCG-ECG, and VCG-ECGsp criteria. (DOCX)

S7 Table Diagnostic performance of Marcos models vs all classic ECG criteria in test set. (DOCX)

S8 Table Subanalysis of diagnostic performance of Marcos criteria in different populations in test set (complement). (DOCX)

S9 Table Comparative performance and design characteristics of published machine learning models for detecting Echo-LVH based on ECG and/or VCG features. (DOCX)

S1 Dataset Anonymized dataset used to reproduce C5.0 models. (CSV)

S1 File R script for training and testing C5.0 models (Marcos criteria). (R)

S1 Text Participant Selection and Cohort Characteristics (Condensed). (DOCX)

S2 Text VCG Data Synthesis and Software Validation. (DOCX)

S3 Text Echocardiographic protocol. (DOCX)
