# Supplementary material for: Clinically interpretable electrovectorcardiographic machine learning criteria for the detection of echocardiographic left ventricular hypertrophy
Source: PLoS One. 2025 Oct 17;20(10):e0334829. doi: 10.1371/journal.pone.0334829 (PMC12533915; doi:10.1371/journal.pone.0334829)
Supplement: S1 Table — (DOCX) [file pone.0334829.s001.docx]

**S1 Table. List of electrocardiogram (ECG) parameters analyzed by the Philips DXL-16 algorithm.**

| **Parameter** | **Units** | **Description** |
| --- | --- | --- |
| **Morphology lead measurements** | | |
| P AMP | mV | P wave amplitude |
| P DUR | msec | P wave duration |
| P AREA | Ashman | P wave area for monophasic P waves or the area of the initial portion of a biphasic P wave |
| P’ AMP | mV | P’ wave amplitude |
| P’ DUR | msec | P' wave duration |
| P’ AREA | Ashman | Area of the terminal portion of a biphasic P wave |
| Q AMP | mV | Q wave amplitude |
| Q DUR | msec | Q wave duration |
| R AMP | mV | R wave amplitude |
| R DUR | msec | R wave duration |
| S AMP | mV | S wave amplitude |
| S DUR | msec | S wave duration |
| R’AMP | mV | R' wave amplitude |
| R’DUR | msec | R' wave duration |
| S’AMP | mV | S' wave amplitude |
| S’DUR | msec | S' wave duration |
| V.A.T. | msec | Ventricular Activation Time is the interval from the onset of the QRS complex to the latest positive peak in the complex, or the latest substantial notch on the latest peak (whichever is later). |
| QRS PPK | mV | Peak-to-peak QRS complex amplitude |
| QRS DUR | msec | QRS complex duration, measured from its onset to the ST segment onset (J point) |
| QRS AREA | Ashman | The area of the QRS complex |
| ST ON | mV | Elevation or depression at the onset (J point) of the ST segment |
| ST MID | mV | Elevation or depression at the midpoint of the ST segment |
| ST 80ms | mV | Elevation or depression of the ST segment 80 ms after the end of the QRS complex (J point) |
| ST END | mV | Elevation or depression at the end of the ST segment |
| ST DUR | msec | ST segment duration |
| ST SLOPE | ° | ST segment slope. Slope is measured in degrees for 25 mm/sec, 1mV/cm scaling, and can range from -90 to +90 degrees. |
| ST SHAPE* | -, V, or ^ | The ST segment shape: - (Straight), V (Concave upward), and ^ (Concave downward) |
| T AMP | mV | T wave amplitude |
| T DUR | msec | T wave duration |
| T AREA | Ashman | T wave area for monophasic T waves or the area of the initial portion of a biphasic T wave |
| T’AMP | mV | T' wave amplitude |
| T’DUR | msec | T' wave duration |
| T’AREA | Ashman | Area of the terminal portion of a biphasic T wave |
| PR INT | msec | Interval from the onset of the P wave to the onset of the QRS complex. |
| PR SEG | msec | Interval from the end of the P wave to the onset of the QRS complex |
| QT INT | msec | Interval from the onset of the QRS complex to the end of the T wave |
| **Derived transverse QRS vector** | | |
| Initial | °, mV | The vector for the initial (first 40 msec) transverse QRS signal |
| Maximum | °, mV | The maximum transverse QRS vector |
| Terminal [ANG_TERM_NUM] | °, mV | The vector from the terminal (last 40 msec) or last part of the transverse QRS signal |
| Rotation | +100 to -100 | The direction of the vector rotation over the entire QRS complex: positive (clockwise rotation), negative (counterclockwise rotation). A larger magnitude indicates a higher confidence in the rotation estimate |
| **Frontal/Horizontal plane axis parameters** | | |
| P | ° | Mean P wave axis |
| I:40 | ° | Initial 40ms QRS complex axis |
| QRS | ° | Mean QRS complex axis |
| T:40 | ° | Terminal 40 msec QRS complex axis |
| ST | ° | Mean ST wave axis |
| T | ° | Mean T wave axis |
| **Global measurements** | | |
| Mean Ventr Rate | BPM | Representative ventricular rate for the entire ECG |
| Mean PR Int | msec | Representative PR interval for the entire ECG |
| Mean PR Seg | msec | Representative PR segment for the entire ECG |
| Mean QRS Dur | msec | Representative QRS duration for the entire ECG |
| Mean QT Int | msec | Representative QT interval for the entire ECG |
| Mean QTc | msec | Representative QT interval adjusted for heart rate |
| QT Dispersion | msec | Difference between the longest and the shortest QT interval in the entire ECG |

This table enumerates various ECG parameters processed through the Philips DXL-16 algorithm. It includes measurements for different waveform components crucial for detailed myocardial activity analysis across a cardiac cycle. The parameters cover morphology, duration, amplitude, and area across the P, QRS, and T phases, as well as specialized intervals and global measurements. Abbreviations: msec (milliseconds), mV (millivolts), Ashman units (40 msec x 0.1 mV), BPM (beats per minute), ° (degrees).
