# Supplementary material for: Clinically interpretable electrovectorcardiographic machine learning criteria for the detection of echocardiographic left ventricular hypertrophy
Source: PLoS One. 2025 Oct 17;20(10):e0334829. doi: 10.1371/journal.pone.0334829 (PMC12533915; doi:10.1371/journal.pone.0334829)
Supplement: S2 Table — (DOCX) [file pone.0334829.s002.docx]

**S2 Table. Diagnostic criteria and corresponding ECG cut-values by various authors.**

| **Criteria** | **Author** | **Cut off value (mV)** | **Formula** |
| --- | --- | --- | --- |
| ECG 1 | Lewis | >1.6 | Sum(Amp_R_I,Amp_S_I,0)+Sum(-Amp_S_III,Amp_R_III,0) |
| ECG 2 | Gubner | >2.5 | Sum(Amp_R_I,-Amp_S_III). |
| ECG 3 | Gubner | >1.5 | Sum(Amp_R_I,0). |
| ECG 4 | Sokolow-Lyon | >1.1 | Sum(Amp_R_Avl,0). |
| ECG 5 | Goldberger | >2.0 | Sum(Amp_R_Avf,0). |
| ECG 6 | Schack | >1.9 | Min(Amp_Q_Avr,Amp_S_Avr). |
| ECG 7 | Romhilt | >1.9 | Max((Amp_R_I-Amp_S_I,0),(Amp_R_II-Amp_S_II,0),(Amp_R_III, Amp_S_III,0),(Amp_R_Avr-Amp_S_Avr,0), (Amp_R_Avl-Amp_S_Avl,0),(Amp_R_Avf-Amp_S_Avf,0)). |
| ECG 8 | Wilson | >2.3 | Sum(-Amp_S_V1,0). |
| ECG 9 | Mazzoleni | >2.5 | Sum(-Amp_S_V2,0). |
| ECG 10 | Sokolow-Lyon | >3.5 | Sum(-Amp_S_V1,0)+Max(Amp_R_V5, Amp_R_V6, 0). |
| ECG 11 | Romhilt | >4.5 | Sum(-Amp_S_V2,0)+Max(Amp_R_V5,Amp_R_V6,0). |
| ECG 12 | Murphy | >3.5 | Max(-Amp_S_V1,-Amp_S_V2,0)+Max(Amp_R_V5,Amp_R_V6,0). |
| ECG 13 | Grant | >4.0 | Max(-Amp_S_V1,-Amp_S_V2,0)+Sum(Amp_R_V6,0). |
| ECG 14 | Grant | >3.5 | Max(Amp_R_V1,Amp_R_V2,Amp_R_V3,Amp_R_V4,Amp_R_V5,Amp_R_V6)+Max(-Amp_S_V1,-Amp_S_V2,-Amp_S_V3,-Amp_S_V4,-Amp_S_V5,-Amp_S_V6). |
| ECG 15 | Holt | >1.0 | Amp_R_V5/Amp_R_V6. |
| ECG 16 | McPhie | >2.6 | Max(Amp_R_V1,Amp_R_V2,Amp_R_V3,Amp_R_V4,Amp_R_V5,Amp_R_V6). |
| ECG 17 | Wolff | >4.5 | Sum(-Amp_S_V2,0)+Max(Amp_R_V4,Amp_R_V5). |
| ECG 18 | Wilson | >3.3 | Amp_R_V5. |
| ECG 19 | Wilson | >2.5 | Amp_R_V6. |
| ECG 20 | Manning | >5.9 | (Sum(Amp_R_Avf,Amp_S_Avf,0)+Sum(Amp_R_V2,Amp_S_V2,0)+Sum(Amp_R_V6-Amp_S_V6,0)). |
| ECG 21 | Casale (Cornell voltage criteria) | >2.8 (men) >2.0 (women) | Sum(-Amp_S_V3,Amp_R_Avl). |
| ECG 22 | Siegel | >17.5 | Sum(-Amp_Q_I,Amp_R_I,-Amp_S_I,-Amp_Q_II,Amp_R_II-Amp_S_II,-Amp_Q_III,Amp_R_III-Amp_S_III,-Amp_Q_Avr,Amp_R_Avr,-Amp_S_Avr, Amp_Q_Avl,Amp_R_Avl,-Amp_S_Avl,-Amp_Q_Avf,Amp_R_Avf,-Amp_S_Avf,-Amp_Q_V1,Amp_R_V1,-Amp_S_V1,-Amp_Q_V2,Amp_R_V2,-Amp_S_V2,-Amp_Q_V3,Amp_R_V3,-Amp_S_V3,-Amp_Q_V4,Amp_R_V4,-Amp_S_V4,-Amp_Q_V5,Amp_R_V5,-Amp_S_V5,-Amp_Q_V6,Amp_R_V6,-Amp_S_V6). |
| ECG 23 | Peguero-Lo Presti | ≥ 2.8 (M) ≥ 2.3 (F) | S_deepest_+SV4 |

S2 Table provides an overview of specific diagnostic criteria using ECG measurements as proposed by different authors. These criteria involve calculated values based on various amplitude measurements from ECG leads. Each criterion is associated with a cut-value threshold that indicates Echo-LVH presence. The table includes authors who developed these criteria and their respective formulas used to compute the ECG values, which are pivotal for clinical interpretations. Abbreviations used in the table includes F (Female), M (male), AMP (Amplitude), SUM (Summation of values), MIN (Minimum value), MAX (Maximum value), and mV (millivolts).
