# Supplementary material for: Clinically interpretable electrovectorcardiographic machine learning criteria for the detection of echocardiographic left ventricular hypertrophy
Source: PLoS One. 2025 Oct 17;20(10):e0334829. doi: 10.1371/journal.pone.0334829 (PMC12533915; doi:10.1371/journal.pone.0334829)
Supplement: S3 Table — (DOCX) [file pone.0334829.s003.docx]

**S3 Table. Parameters of vectorcardiography (VCG) and their clinical descriptions.**

| **VCG Parameter (reference)** | **Unit** | **Description** |
| --- | --- | --- |
| Area [20, 21] | μVs | The 3D area under the curve from the onset and offset of each loop. |
| Geometric area vector [23] | None | Resultant vector of normals to the triangles formed by consecutive loop vectors. |
| Planarity index [23] | None | The ratio of the magnitude of the geometrical vector area to the entire loop area. |
| Azimuth [23] | ° | The vector’s angle between the xz-plane projection and the positive x-axis, directed towards the -/+ z-axis. |
| Elevation [23] | ° | The angle between the vector and the positive y-axis in the range of -180° to +180°. |
| Roundness index [24] | None | The ratio between the loop area to the square of the maximum loop vector passing through its origin. |
| Thickness [18] | μV | The distance between two parallel fitting planes encompassing the entire horizontal loop. |
| Dihedral angle variability [18] | ° | Dihedral angle variability across the entire loop measured by the RMSSD. |
| Mean di angle* [18] | ° | Mean dihedral angle measures the overall mean planar changes in the loop over time. |
| Dihedral angle SD [18] | ° | Di angle SD across the entire loop; this measures the dispersion of the planar change in the loop over time. |
| Dihedral angle range [18] | ° | Dihedral angle range measures the dispersion of the planar change in the loop over time. |
| Dihedral angle* [18] | ° | The angular relationship between two intersecting unit planes. |
| Rotational angle var [18] | ° | Rotational angle variability across the entire loop measured by the RMSSD. |
| Mean Rotational angle* [18] | ° | Mean rotational angle across the entire loop. |
| Rotational angle SD [18] | ° | Rotational angle standard deviation across the entire loop. |
| Rotational angle range* [18] | ° | Rotational angle range across the entire loop. |
| Rotational angle [18] | ° | The angle between two consecutive loop vectors obtained by RMSSD. |
| Velocity* [19] | μV/ms | The distance traveled by the heart vector along the loop in three-dimensional space. |
| Max velocity [19] | μV/ms | Maximum loop velocity. |
| Min velocity [19] | μV/ms | Minimum loop velocity. |
| Velocity variability [19] | μV/ms | Velocity variability across the entire loop. |
| Curvature* [19] | μV^−1^ | Measures the VCG loop deviation from a straight line at a given point. |
| Orbital frequency* [19] | ms^−1^ | A scalar measure of the rotation rate of the loop vector, calculated as the product of velocity and curvature. |
| Magnitude* [18] | μV | Maximum vector passing through the loop. |
| Lag-1 and -1/10 Rot/Di [18] | ° | Autocorrelation coefficients (ACC) assesses the correlation between a time series and its lagged version. |
| SAI QRS loop [21, 34, 35] | μVs | The arithmetic sum of the area under the QRS loop on three orthogonal leads (x, y, and z) |
| SAI QRS-T loop [34, 35, 36] | μVs | The arithmetic sum of areas under the QRS-T loop on three orthogonal leads (x, y, and z) |
| SAI J-T loop [34, 35, 37] | μVs | The arithmetic sum of areas under the J-T loop on three orthogonal leads (x, y, and z) |
| SVG [34, 36, 37, 38] | mV | 3D direction and magnitude of electrical activity variation, based on the area beneath the QRS-T interval. |
| SVG azimuth [35] | ° | Azimuth angle of the SVG. |
| SVG elevation [35] | ° | Elevation angle of the SVG. |

S3 Table summarizes key parameters of vectorcardiography (VCG), detailing units and descriptions for each. VCG parameters were obtained from the P loop, QRS loop, and T loop using Kors, Downer, QLSV, and PLSV matrices, both with and without the use of a Fourier filter. These VCG parameters include geometrical and temporal aspects of the cardiac vector loop such as area, angles, velocity, and variability. These parameters provide insights into the heart's electrical activity in three-dimensional space, aiding in the diagnosis of left ventricular hypertrophy. The table also describes the dihedral and rotational angles related to the heart's vector movement, along with their mean values, variability, and standard deviations, providing comprehensive data for clinical assessments. *****Measurements were also calculated for each tenth part of each loop. Abbreviations include Lag, RMSSD (root mean squared of successive differences, SAI: sum absolute integral, SVG: spatial ventricular gradient, SD: standard deviation.
