# Supplementary material for: Clinically interpretable electrovectorcardiographic machine learning criteria for the detection of echocardiographic left ventricular hypertrophy
Source: PLoS One. 2025 Oct 17;20(10):e0334829. doi: 10.1371/journal.pone.0334829 (PMC12533915; doi:10.1371/journal.pone.0334829)
Supplement: S4 Table — (DOCX) [file pone.0334829.s004.docx]

**S4 Table. Low-prevalence comorbidities and conduction disorders in the training and test cohorts.**

|  | **Total (n=664)** | **Training set (n=460)** | **Testing set (n=204)** | **p-value** |
| --- | --- | --- | --- | --- |
| Pacemaker (n/N, %)* | 6/373 (1.6) | 6/266 (2.3) | 0/107 (0) | 0.19 |
| Chronic obstructive pulmonary disease (n/N, %)* | 8/482 (1.7) | 4/337 (1.2) | 4/145 (2.8) | 0.25 |
| Reumathoid arthirtis (n/N, %)* | 9/482 (1.9) | 5/337 (1.5) | 4/145 (2.8) | 0.46 |
| Pulmonary embolism (n/N, %) | 12/482 (2.5) | 6/337 (1.8) | 6/145 (4.1) | 0.13 |
| Sick sinus syndrome (n/N, %)* | 5/482 (1) | 4/337 (1.2) | 1/145 (0.7) | 0.62 |
| Aortic stenosis (n/N, %) | 25/482 (5.2) | 17/337 (5) | 8/145 (5.5) | 0.83 |
| Supraventricular tachycardia (n/N, %)* | 15/482 (3.1) | 11/337 (3.3) | 4/145 (2.8) | 0.51 |
| Obstructive sleep apnea syndrome (n/N, %)* | 5/482 (1) | 3/337 (0.9) | 2/145 (1.4) | 0.64 |
| Peripheral artery disease (n/N, %)* | 18/482 (3.7) | 15/337 (4.5) | 3/145 (2.1) | 0.30 |
| Pulmonary hypertension (n/N, %)* | 7/482 (1.5) | 5/337 (1.5) | 2/145 (1.4) | 0.65 |
| Hypothyroidism (n/N, %) | 45/482 (9.3) | 26/337 (7.7) | 19/145 (13.1) | 0.06 |

*P values were calculated using Fisher’s exact test for conditions with expected cell counts <5. Chi-square test was applied in other comparisons. Total sample size varies per row due to missing data for some conditions. Denominators are indicated for each row as n/N (%).
