# Supplementary material for: Clinically interpretable electrovectorcardiographic machine learning criteria for the detection of echocardiographic left ventricular hypertrophy
Source: PLoS One. 2025 Oct 17;20(10):e0334829. doi: 10.1371/journal.pone.0334829 (PMC12533915; doi:10.1371/journal.pone.0334829)
Supplement: S5 Table — (DOCX) [file pone.0334829.s005.docx]

**S5 Table. Dimensionality reduction of VCG and ECG parameters with Lasso regression**

| **Data** | **Parameters [number of predictors]** | **Best lambda** | **AUC [Train]** | **AUC [Test]** |
| --- | --- | --- | --- | --- |
| **VCG** | **Global Measurements:** RangeAngR D, Lag 1Ro D, QRSA K, GAV K, Elevation SVG Q. **Auricular (P loop):** POrbFrqD6, POrbFrqP7, PCurvD5, PCurvK6 lag, PCurvQ4 lag, PRangeDi10K3 lag, PRangeRot10Q2 lag. **Ventricular Depolarization (QRS loop):** elev P, Elevation SVG P, DesvEstAngk Kf P, QRSMagP1, QRSMeanDi10K6, QRSMeanRot10K1, QRSMeanRot10P9, QRSRangeRot10Q2, QRSRangeRot10P9, QRSOrbVelP8 lag, QRSMagP9 lag, QRSMagPf8 lag. **Ventricular Repolarization (T loop):** RangeAngR Q T, Lag 1/10Di Df T, TOrbVelQ5, TMagK2, TMagD2 lag, TMagD10 lag, TMagDf3 lag, TMeanRot10D8 lag, TMeanRot10P6. **[33]** | 0.053 | 0.82 | 0.74 |
| **ECG** | **Global measurements / intervals:** PR INT III, PR SEG V3, PR SEG V6, QT INT V2. **Auricular (P wave):** AMP P II, DUR P V4 **Ventricular Depolarization (QRS complex):** QRS DUR AVF**,** DUR R AVL, DUR R V3, QRS PPK V4, AMP S V3, AMP S V6, VAT AVL, VAT V3, ECG 21 (Cornell voltage) **ST segment:** ST MID II, ST 80mS AVR, ST DUR I, ST SHAPE V4, ST ANG FRONT **Ventricular Repolarization (T wave):** T ANG FRONT, T ANG HOR, T AREA I, T AREA V5 **Transverse QRS vector / axis features:** ANG IN GRAD, ANG TERM NUM **[26]** | 0.03 | 0.81 | 0.7 |

The 33 VCG and 26 ECG predictors selected by Lasso regression include spatial magnitudes (e.g., QRSMagP1), angular measurements (e.g., RangeAngR D), curvature (e.g., PCurvD5), velocity (e.g., TOrbVelQ5), and timing intervals (e.g., PR INT III), measured across P, QRS, or T waves. VCG parameters were derived using four vector transformation matrices: P = PLSV, Q = QLSV, K = Kors, and D = Downer. Full variable definitions are available in Supplementary Tables S1 (ECG) and S3 (VCG). Interestingly, none of the other state-of-the-art criteria or their VDP versions were selected by the Lasso Regression model except for the Cornell voltage and components from Peguero-Lo Presti.
