# Supplementary material for: Clinically interpretable electrovectorcardiographic machine learning criteria for the detection of echocardiographic left ventricular hypertrophy
Source: PLoS One. 2025 Oct 17;20(10):e0334829. doi: 10.1371/journal.pone.0334829 (PMC12533915; doi:10.1371/journal.pone.0334829)
Supplement: S6 Table — (DOCX) [file pone.0334829.s006.docx]

**S6 Table. Ten-fold cross validation for the proposed Marcos VCG, VCG-ECG, and VCG-ECGsp criteria.**

| **Marcos VCG** | | | | | | | |
| --- | --- | --- | --- | --- | --- | --- | --- |
| **Fold** | **Acc** | **95% CI Lower** | **95% CI Upper** | **Se** | **Sp** | **PPV** | **NPV** |
| 1 | 80.4 | 66.1 | 90.6 | 63.2 | 92.6 | 85.7 | 78.1 |
| 2 | 71.7 | 56.5 | 84 | 47.4 | 88.9 | 75 | 70.6 |
| 3 | 71.7 | 56.5 | 84 | 63.2 | 77.8 | 66.7 | 75 |
| 4 | 80 | 65.4 | 90.4 | 63.2 | 92.3 | 85.7 | 77.4 |
| 5 | 78.3 | 63.6 | 89.1 | 57.9 | 92.6 | 84.6 | 75.8 |
| 6 | 65.2 | 49.8 | 78.6 | 47.4 | 77.8 | 60 | 67.7 |
| 7 | 78.3 | 63.6 | 89.1 | 57.9 | 92.6 | 84.6 | 75.8 |
| 8 | 78.3 | 63.6 | 89.1 | 57.9 | 92.6 | 84.6 | 75.8 |
| 9 | 78.3 | 63.6 | 89.1 | 63.2 | 88.9 | 80 | 77.4 |
| 10 | 78.7 | 64.3 | 89.3 | 75 | 81.5 | 75 | 81.5 |
| **Mean** | 76.09 | 61.3 | 87.33 | 59.63 | 87.76 | 78.19 | 75.51 |
| **Marcos VCG-ECG** | | | | | | | |
| **Fold** | **Acc** | **95% CI Lower** | **95% CI Upper** | **Se** | **Sp** | **PPV** | **NPV** |
| 1 | 76.1 | 61.2 | 87.4 | 73.7 | 77.8 | 70 | 80.8 |
| 2 | 80.4 | 66.1 | 90.6 | 89.5 | 74.1 | 70.8 | 90.9 |
| 3 | 78.3 | 63.6 | 89.1 | 84.2 | 74.1 | 69.6 | 87 |
| 4 | 77.8 | 62.9 | 88.8 | 73.7 | 80.8 | 73.7 | 80.8 |
| 5 | 71.7 | 56.5 | 84 | 68.4 | 74.1 | 65 | 76.9 |
| 6 | 73.9 | 58.9 | 85.7 | 57.9 | 85.2 | 73.3 | 74.2 |
| 7 | 76.1 | 61.2 | 87.4 | 73.7 | 77.8 | 70 | 80.8 |
| 8 | 78.3 | 63.6 | 89.1 | 84.2 | 74.1 | 69.6 | 87 |
| 9 | 71.7 | 56.5 | 84 | 73.7 | 70.4 | 63.6 | 79.2 |
| 10 | 83 | 69.2 | 92.4 | 95 | 74.1 | 73.1 | 95.2 |
| **Mean** | 76.73 | 61.97 | 87.85 | 77.4 | 76.25 | 69.87 | 83.28 |
| **Marcos VCG-ECGsp** | | | | | | | |
| **Fold** | **Acc** | **95% CI Lower** | **95% CI Upper** | **Se** | **Sp** | **PPV** | **NPV** |
| 1 | 84.8 | 71.1 | 93.7 | 73.7 | 92.6 | 87.5 | 83.3 |
| 2 | 80.4 | 66.1 | 90.6 | 68.4 | 88.9 | 81.3 | 80 |
| 3 | 78.3 | 63.6 | 89.1 | 73.7 | 81.5 | 73.7 | 81.5 |
| 4 | 71.1 | 55.7 | 83.6 | 52.6 | 84.6 | 71.4 | 70.9 |
| 5 | 73.9 | 58.9 | 85.7 | 63.2 | 81.5 | 70.6 | 75.9 |
| 6 | 73.9 | 58.9 | 85.7 | 52.6 | 88.9 | 76.9 | 72.7 |
| 7 | 67.4 | 51.9 | 80.5 | 52.6 | 77.8 | 62.5 | 70 |
| 8 | 69.6 | 54.3 | 82.3 | 57.9 | 77.8 | 64.7 | 72.4 |
| 9 | 76.1 | 61.2 | 87.4 | 63.2 | 85.2 | 75 | 76.7 |
| 10 | 78.7 | 64.3 | 89.3 | 75 | 81.5 | 75 | 81.5 |
| **Mean** | 75.42 | 60.6 | 86.79 | 63.29 | 84.03 | 73.86 | 76.49 |
