# Supplementary material for: Clinically interpretable electrovectorcardiographic machine learning criteria for the detection of echocardiographic left ventricular hypertrophy
Source: PLoS One. 2025 Oct 17;20(10):e0334829. doi: 10.1371/journal.pone.0334829 (PMC12533915; doi:10.1371/journal.pone.0334829)
Supplement: S7 Table — (DOCX) [file pone.0334829.s007.docx]

**S7 Table. Diagnostic performance of Marcos models vs all classic ECG criteria in test set**

| **Criteria/Author** | **Cut-off value (mV)** | **Predictors (#)** | **Acc (95%CI)** | Δ **Acc (%)** | **Se (%)** | Δ **Se (%)** | **F1 score** | **Sp (%)** | **PPV (%)** | **NPV (%)** |
| --- | --- | --- | --- | --- | --- | --- | --- | --- | --- | --- |
| Marcos VCG-ECG (test set) | NA | 6 | 75.5 (69-81.2) | Baseline | 73.1 | Baseline | 0.731 | 77.5 | 73.1 | 77.5 |
| Marcos VCG-ECGsp (test set) | NA | 6 | 72.6 (65.9-78.6) | -2.9 | 62.4 | -10.7 | 0.674 | 81.1 | 73.4 | 72 |
| Marcos VCG (test set) | NA | 5 | 72.1 (65.4-78.1) | -3.4 | 55.9 | -17.2 | 0.645 | 85.6 | 76.5 | 69.9 |
| ECG 1 (Lewis) | >1.6 | 4 | 56.4 (49.27-63.29) | -19.1 | 14 | -59.1 | 0.226 | 91.9 | 59.1 | 56 |
| ECG 2 (Gubner) | >2.5 | 2 | 54.4 (47.31-61.38) | -21.1 | 0 | -73.1 | NA | 100 | NA | 54.4 |
| ECG 3 (Gubner) | >1.5 | 1 | 55.4 (48.29-62.34) | -20.1 | 4.3 | -68.8 | 0.08 | 98.2 | 66.7 | 55.1 |
| ECG 4 (Sokolow-Lyon) | >1.1 | 1 | 57.8 (50.75-64.71) | -17.7 | 12.9 | -60.2 | 0.218 | 95.5 | 70.6 | 56.7 |
| ECG 5 (Goldberger) | >2 | 1 | 53.9 (46.82-60.91) | -21.6 | 0 | -73.1 | NA | 99.1 | 0 | 54.2 |
| ECG 6 (Schack) | >1.9 | 2 | 54.4 (47.31-61.38) | -21.1 | 0 | -73.1 | NA | 100 | NA | 54.4 |
| ECG 7 (Romhilt) | >1.9 | 12 | 55.3 (48.1-62.4) | -20.2 | 3.3 | -69.8 | 0.063 | 100 | 100 | 54.6 |
| ECG 8 (Wilson) | >2.3 | 1 | 54.9 (47.8-61.86) | -20.6 | 1.1 | -72 | 0.021 | 100 | 100 | 54.7 |
| ECG 9 (Mazzoleni) | >2.5 | 1 | 54.9 (47.8-61.86) | -20.6 | 2.2 | -70.9 | 0.042 | 99.1 | 66.7 | 54.7 |
| ECG 10 (Sokolow-Lyon) | >3.5 | 2 | 55.4 (48.29-62.34) | -20.1 | 3.2 | -69.9 | 0.061 | 99.1 | 75 | 55 |
| ECG 11 (Romhilt) | >4.5 | 3 | 53.9 (46.82-60.91) | -21.6 | 0 | -73.1 | NA | 99.1 | 0 | 54.2 |
| ECG 12 (Murphy) | >3.5 | 4 | 56.9 (49.76-63.76) | -18.6 | 8.6 | -64.5 | 0.153 | 97.3 | 72.7 | 56 |
| ECG 13 (Grant) | >4.0 | 3 | 55.4 (48.29-62.34) | -20.1 | 3.2 | -69.9 | 0.061 | 99.1 | 75 | 55 |
| ECG 14 (Grant) | >3.5 | 12 | 59.8 (52.73-66.59) | -15.7 | 17.2 | -55.9 | 0.28 | 95.5 | 76.2 | 57.9 |
| ECG 15 (Holt) | >1 | 2 | 48.3 (41.23-55.38) | -27.2 | 83.9 | 10.8 | 0.597 | 18.2 | 46.4 | 57.1 |
| ECG 16 (McPhie) | >2.6 | 6 | 54.9 (47.8-61.86) | -20.6 | 3.2 | -69.9 | 0.06 | 98.2 | 60 | 54.8 |
| ECG 17 (Wolff) | >4.5 | 3 | 54.4 (47.31-61.38) | -21.1 | 1.1 | -72 | 0.021 | 99.1 | 50 | 54.5 |
| ECG 18 (Wilson) | >3.3 | 1 | 53.7 (46.58-60.7) | -21.8 | 0 | -73.1 | NA | 99.1 | 0 | 54 |
| ECG 19 (Wilson) | >2.5 | 1 | 54.4 (47.31-61.38) | -21.1 | 1.1 | -72 | 0.021 | 99.1 | 50 | 54.5 |
| ECG 20 (Manning) | >5.9 | 6 | 54.9 (47.8-61.86) | -20.6 | 1.1 | -72 | 0.021 | 100 | 100 | 54.7 |
| ECG 21 (Cornell voltage) | >2.8 (M) / >2 (F) | 2 | 62.3 (55.22-68.93) | -13.2 | 21.5 | -51.6 | 0.341 | 96.4 | 83.3 | 59.4 |
| ECG 22 (Siegel) | >17.5 | 36 | 58.8 (51.74-65.65) | -16.7 | 14 | -59.1 | 0.236 | 96.4 | 76.5 | 57.2 |
| ECG 23 (Peguero-Lo Presti) | ≥2.8 (M) /≥2.3 (F) | 13 | 63.7 (56.7-70.3) | -11.8 | 30.1 | -43 | 0.433 | 91.9 | 75.7 | 61.1 |

This table compares the diagnostic performance of the Marcos models against twenty-three ECG criteria. It includes authors, cut-off values, the number of variables used, and key metrics like accuracy, delta accuracy, sensitivity, specificity, positive predictive value (PPV), and negative predictive value (NPV). All Marcos Criteria demonstrate improved diagnostic metrics compared to all ECG criteria. The two ECG criteria and this study's models with the best predictive capacity for Echo-LVH are highlighted in gray. Abbreviations: Acc: accuracy, F: female, M: male, Se: Sensitivity, Sp: Specificity, PPV: Positive Predictive Value, NPV: Negative Predictive Value. Δ values represent the difference between the listed ECG criterion and the baseline Marcos VCG-ECG metric. For example, ΔSe = (Marcos VCG-ECG Se) − (ECG Se).
