# Supplementary material for: Clinically interpretable electrovectorcardiographic machine learning criteria for the detection of echocardiographic left ventricular hypertrophy
Source: PLoS One. 2025 Oct 17;20(10):e0334829. doi: 10.1371/journal.pone.0334829 (PMC12533915; doi:10.1371/journal.pone.0334829)
Supplement: S8 Table — (DOCX) [file pone.0334829.s008.docx]

**S8 Table. Subanalysis of diagnostic performance of Marcos criteria in different populations in test set (complement).**

|  |  | **Marcos VCG** | **Marcos VCG-ECG** | **Cornell voltage** | **Peguero-Lo Presti** |
| --- | --- | --- | --- | --- | --- |
| **Female** (n=91)  Echo-LVH (n=48, 52.7%) | **Acc (95%CI)** | 74.7 (64.5-83.3)  60.4  90.7  87.9  67.2 | 74.7 (64.5-83.3)  70.8  79.1  79.1  79.8 | 57.1 (46.3-67.5)  27.1  90.7  76.5  52.7 | 60.4 (49.6-70.5)  31.3  93  83.3  54.8 |
|  | **Se (%)** |  |  |  |  |
|  | **Sp (%)** |  |  |  |  |
|  | **PPV (%)** |  |  |  |  |
|  | **NPV (%)** |  |  |  |  |
| **Male** (n=113)  Echo-LVH (n=45, 39.8%) | **Acc (95%CI)** | 69.9 (60.6-78.2)  51.1  82.3  65.7  71.8 | 76.1 (67.2-83.6)  75.6  76.5  68  82.5 | 66.4 (56.9-75)  15.6  100  100  64.2 | 67.3 (57.8-75.8)  28.9  92.7  72.2  66.3 |
|  | **Se (%)** |  |  |  |  |
|  | **Sp (%)** |  |  |  |  |
|  | **PPV (%)** |  |  |  |  |
|  | **NPV (%)** |  |  |  |  |
| **>60 years** (n=127)  Echo-LVH (n=63, 49.6%) | **Acc (95%CI)** | 69.3 (60.5-77.2)  52.4  85.9  78.6  64.7 | 71.7 (63-78.3)  74.6  68.8  70.2  73.3 | 57.6 (48.4-66.2)  20.6  93.8  76.5  54.6 | 60.6 (51.6-69.2)  27  93.8  80.9  56.6 |
|  | **Se (%)** |  |  |  |  |
|  | **Sp (%)** |  |  |  |  |
|  | **PPV (%)** |  |  |  |  |
|  | **NPV (%)** |  |  |  |  |
| **≤60 years** (n=77)  Echo-LVH (n=30, 39%) | **Acc (95%CI)** | 76.6 (65.6-85.5)  63.3  85.1  73.1  78.4 | 81.8 (71.4-89.7)  70  89.4  80.8  82.4 | 70.1 (58.6-80)  23.3  100  100  67.1 | 70.1 (58.6-80)  36.7  91.5  73.3  69.4 |
|  | **Se (%)** |  |  |  |  |
|  | **Sp (%)** |  |  |  |  |
|  | **PPV (%)** |  |  |  |  |
|  | **NPV (%)** |  |  |  |  |
| **BMI ≥30 kg/m^2^** (n=54)  Echo-LVH (n=29, 53.7%) | **Acc (95%CI)** | 64.8 (50.6-77.3)  44.8  88  81.3  57.9 | 72.2 (58.4-83.5)  72.4  72  75  69.2 | 55.6 (41.4-69.1)  17.2  100  100  51 | 57.4 (43.2-70.8)  27.6  92  80  52.3 |
|  | **Se (%)** |  |  |  |  |
|  | **Sp (%)** |  |  |  |  |
|  | **PPV (%)** |  |  |  |  |
|  | **NPV (%)** |  |  |  |  |
| **BMI <30 kg/m^2^** (n=150)  Echo-LVH (n=64, 42.7%) | **Acc (95%CI)** | 74.7 (66.9-81.1)  60.9  84.9  75  74.5 | 76.7 (69.1-83.2)  73.4  79.1  72.3  80 | 64.7 (56.5-72.3)  23.4  95.4  78.9  62.6 | 66.7 (58.5-74.1)  31.3  93  76.9  64.5 |
|  | **Se (%)** |  |  |  |  |
|  | **Sp (%)** |  |  |  |  |
|  | **PPV (%)** |  |  |  |  |
|  | **NPV (%)** |  |  |  |  |
| **Hypertension +** (n=89)  Echo-LVH (n=53, 59.6%) | **Acc (95%CI)** | 66.3 (55.5-76)  50.9  88.9  87.1  55.2 | 71.2 (61.4-80.9)  75.5  66.7  76.9  64.9 | 51.7 (40.8-62.4)  22.6  94.4  85.7  45.3 | 58.4 (47.5-68.8)  35.9  91.7  86.4  49.3 |
|  | **Se (%)** |  |  |  |  |
|  | **Sp (%)** |  |  |  |  |
|  | **PPV (%)** |  |  |  |  |
|  | **NPV (%)** |  |  |  |  |
| **Hypertension -** (n=57)  Echo-LVH (n=23, 40.4%) | **Acc (95%CI)** | 77.2 (64.2-87.3)  60.9  88.2  77.8  76.9 | 82.5 (70.1-91.3)  82.6  82.4  76  87.5 | 66.7 (52.9-78.6)  17.4  100  100  64.2 | 64.9 (51.1-77.1)  26.1  91.2  66.7  64.6 |
|  | **Se (%)** |  |  |  |  |
|  | **Sp (%)** |  |  |  |  |
|  | **PPV (%)** |  |  |  |  |
|  | **NPV (%)** |  |  |  |  |
| **Echo-IHD +** (n=42)  Echo-LVH (n=21, 50%) | **Acc (95%CI)** | 78.6 (63.2-89.7)  66.7  90.5  87.5  73.1 | 69.1 (52.9-82.4)  81  57.1  65.4  75 | 61.9 (45.6-76.4)  23.8  100  100  56.8 | 71.4 (55.4-84.3)  42.9  100  100  63.6 |
|  | **Se (%)** |  |  |  |  |
|  | **Sp (%)** |  |  |  |  |
|  | **PPV (%)** |  |  |  |  |
|  | **NPV (%)** |  |  |  |  |
| **Echo-IHD -** (n=162)  Echo-LVH (n=72, 44.4%) | **Acc (95%CI)** | 70.4 (62.7-77.3)  52.8  84.4  73.1  69.1 | 77.2 (69.9-83.4)  70.8  82.2  76.1  77.9 | 62.4 (54.4-69.8)  20.8  95.6  78.9  60.1 | 62.4 (54.4-69.8)  26.4  91.1  70.4  60.7 |
|  | **Se (%)** |  |  |  |  |
|  | **Sp (%)** |  |  |  |  |
|  | **PPV (%)** |  |  |  |  |
|  | **NPV (%)** |  |  |  |  |

This table presents a detailed subanalysis of the Marcos criteria's performance across various patient populations, including sex, age groups, obesity status, hypertension status and Echo-IHD. It compares the accuracy, sensitivity, specificity, PPV, and NPV of the Marcos criteria against traditional criteria like Cornell voltage and Peguero-Lo Presti in these subgroups. This analysis highlights the effectiveness of the Marcos criteria in diverse clinical scenarios. The model with the highest accuracy for each category is highlighted in gray. Abbreviations: Acc: accuracy, BMI: body mass index, Echo-IHD: echocardiographic ischemic heart disease, Se: Sensitivity, Sp: Specificity, PPV: Positive Predictive Value, NPV: Negative Predictive Value.
