# Supplementary material for: Clinically interpretable electrovectorcardiographic machine learning criteria for the detection of echocardiographic left ventricular hypertrophy
Source: PLoS One. 2025 Oct 17;20(10):e0334829. doi: 10.1371/journal.pone.0334829 (PMC12533915; doi:10.1371/journal.pone.0334829)
Supplement: S9 Table — (DOCX) [file pone.0334829.s009.docx]

**S9 Table. Comparative performance and design characteristics of published machine learning models for detecting Echo-LVH based on ECG and/or VCG features.**

| **Ref.** | **First Author / Year** | **Country** | **Population** | **LVMI Threshold** | **Variables Used** | **Model Type** | **# Predictors** | **AUC** | **Accuracy (%)** | **Sensitivity (%)** | **Specificity (%)** | **F1-score** | **Key Limitations / Notes** |
| --- | --- | --- | --- | --- | --- | --- | --- | --- | --- | --- | --- | --- | --- |
| [77] | Sabovčik et al., 2021 | Belgium | General population (n NR) | Same | Clinical + ECG | LR, RF, XGBoost, AdaBoost, SVM | 67 | 0.771–0.785 | NR | 64–74.7 | 63–70.6 | 0.458–0.478 | F1-score among lowest reported; limited specificity |
| [78] | Agelaki et al., 2023 | Greece | >30 yrs, no CVD (n NR) | Same | 6 clinical + 26 ECG | Random Forest | 32 | 0.87 | 88 | 44 | 97 | NR | Excellent specificity; no comparison with ECG criteria |
| [79] | Sammami et al., 2022 | Netherlands | Hospitalized (n = 28,954) | Same | Echo + ECG | XGBoost | 41 | NR | NR | 32 | 99 | NR | Highest specificity (99%); lowest sensitivity (32%); very low LVH prevalence (0.8%) |
| [80] | Lim et al., 2022 | South Korea | Military men (n = 17,310) | Same | Demographic + clinical + ECG | RF, LR, GLMNet, GBM | 45 | 0.8–0.873 | NR | 70.1–87.9 | 67.6–86.5 | 0.035–0.073 | Highest AUC range; lowest F1-scores; very low LVH prevalence (0.82%) |
| [81] | Zhao et al., 2022 | China | Hospital-based (n = 1,863) | Same | Echo M-mode + ECG segments | CNN-LSTM | 36,350 segments | 0.578–0.677 | NR | 47–72 | 57–71 | 0.53–0.68 | Moderate sensitivity; separate AUCs for concentric/eccentric LVH by sex |
| [82] | Liu CW et al., 2021 | Taiwan | 90.5% male (n NR) | Same | 24 ECG signal features | DT, K-means, BPN | 24 | 0.59–0.96 | 59–96.1 | 79.9–96.6 | 38–95.6 | **0.961** (highest) | Highest F1-score reported; beat segmentation improved all metrics |
| [83] | Lin & Liu, 2020 | Taiwan | Military men (n NR) | Different | ECG + clinical | SVM | NR | 0.871 | 77.9 | **92.6** (highest) | 75.1 | 0.322 | Highest sensitivity among all studies; low LVH prevalence (6.5%) |
| [84] | Kwon et al., 2021 | South Korea | Adults >18 yrs (n NR) | Different | Demographic + ECG | ENN, CNN, DNN, LR, RF | NR | 0.81–0.868 | 84.6–86.6 | 36.4–49.6 | **93.6** | NR | Matched ECG specificity; strong accuracy |
| [85] | Kokubo et al., 2021 | Japan | Hospital-based >18 yrs (n = 18,763) | Different | ECG parameters | CNN, DNN, ENN, LR, RF | NR | 0.716–0.784 | 68.3–73.2 | 39.3–60.5 | 81.3–90.7 | NR | High specificity (up to 95.2%); broad ML comparisons |
| [86] | Liu CM et al., 2021 | Taiwan/Japan | Veterans 20–60 yrs (n = 28,745) | Different | Demographic + 2D ECG signals | DNN | NR | NR | 68 | 90.3 | 69.3 | NR | External validation showed high sensitivity with modest specificity |
| [87] | Kataoka et al., 2021 | Japan | All ages (n = 5,935) | Same | ECG + VCG | Random Forest | 46 | NR | 90.4 (VCG), 86.7 (ECG) | NR | NR | **0.903–0.904 (VCG)** | Highest F1-score after Liu CW; used VCG+ECG; high performance in both models |
