# Supplementary material for: Clinically interpretable electrovectorcardiographic machine learning criteria for the detection of echocardiographic left ventricular hypertrophy
Source: PLoS One. 2025 Oct 17;20(10):e0334829. doi: 10.1371/journal.pone.0334829 (PMC12533915; doi:10.1371/journal.pone.0334829)
Supplement: S1 Text — (DOCX) [file pone.0334829.s012.docx]

**S1 Text. Participant Selection and Cohort Characteristics (Condensed)**

A total of 7,567 consecutive patients who underwent both ECG and transthoracic echocardiography were initially screened. The following exclusion criteria were applied:

- Age <18 years (n = 2,300)
- Incomplete ECG or Echo data (n = 3,710)
- Cardiac conditions interfering with ECG-VCG interpretation (n = 885), including:
  - QRS duration >120 ms
  - Fusion or pacemaker rhythms
  - Hypertrophic or dilated cardiomyopathy
  - Ventricular dilation or asymmetric hypertrophy
  - Acute ischemia (e.g., ST changes, elevated cardiac enzymes)
  - Septal defects, preexcitation, bundle branch blocks, hemiblocks
  - Tachycardia >110 bpm, ICU status, or recent cardiotomy (<3 months)

Additionally, eight ECG files were excluded due to data corruption.

After applying all criteria, 664 patients were included in the final analysis.
