# Supplementary material for: Clinically interpretable electrovectorcardiographic machine learning criteria for the detection of echocardiographic left ventricular hypertrophy
Source: PLoS One. 2025 Oct 17;20(10):e0334829. doi: 10.1371/journal.pone.0334829 (PMC12533915; doi:10.1371/journal.pone.0334829)
Supplement: S2 Text — (DOCX) [file pone.0334829.s013.docx]

**S2 Text. VCG Data Synthesis and Software Validation**

ECG tracings were digitized from vector images (Inkscape v1.0.2), producing 600 time points across eight leads (I, II, V1–V6). Vectorcardiogram (VCG) signals were then synthesized using Marcos Software (MATLAB), applying matrix transformations:

- Inverse Downer
- Kors regression
- QLSV
- PLSV

Signal refinement included Fourier filtering and intra-loop dynamics. The software allowed manual annotation of wave onset/offset and loop segmentation (P, QRS, T) for feature extraction.

Each loop was divided into 10 segments to assess intra-loop variability (e.g., velocity), and lag variables (Lag = 1) were calculated to capture temporal autocorrelation. This process yielded 3,360 VCG features per patient (excluding lags). Output was exported as structured CSV files.
