# Supplementary material for: Clinically interpretable electrovectorcardiographic machine learning criteria for the detection of echocardiographic left ventricular hypertrophy
Source: PLoS One. 2025 Oct 17;20(10):e0334829. doi: 10.1371/journal.pone.0334829 (PMC12533915; doi:10.1371/journal.pone.0334829)
Supplement: S3 Text — (DOCX) [file pone.0334829.s014.docx]

**S3 Text. Echocardiographic protocol**

Transthoracic echocardiograms were performed using 2D ECG-guided M-mode imaging on Philips EPIQ7 and IE33 systems, following European and American guidelines. This technique offers accurate orientation for LV wall measurements in patients with normal ventricular geometry (e.g., hypertension), making it appropriate for our study goals.

Three cardiologists conducted the exams, achieving high inter-rater agreement (κ = 0.91). The following diastolic measurements were obtained:

- Left ventricular internal diameter (LVID)
- Interventricular septal thickness (IVST)
- Posterior wall thickness (LVPWT)

Left ventricular mass (LVM) was calculated using the Devereux formula:

$$LVM=0.8 \times1.04 {((LVID+LVPWT+IVST)}^{3}- \left( LVID \right)^{3})+0.6g$$

LVM was indexed to body surface area (BSA) to obtain the LV mass index (LVMI):

$$LVMI=\frac{LVM}{BSA}$$

**Echo-LVH was defined** as LVMI >115 g/m² for men and >95 g/m² for women. Severity thresholds were:

- Mild: 103–116 (men), 89–100 (women)
- Moderate: 117–130 (men), 101–112 (women)
- Severe: >130 (men), >112 (women) [33]

**LV geometry** was classified by combining LVMI and relative wall thickness (RWT):

$$RWT = \frac{2 \times LVPWT}{LVID}$$

- Normal geometry: normal LVMI + RWT < 0.42
- Concentric remodeling: normal LVMI + RWT ≥ 0.42
- Concentric hypertrophy: high LVMI + RWT ≥ 0.42
- Eccentric hypertrophy: high LVMI + RWT < 0.42

**Ischemic heart disease** on echo was defined as segmental akinesia/hypokinesia in coronary territories and was recorded independently of ECG Q waves.
